# Hemorrhagic Complications of Minimally Invasive Urological Surgeries, Treated with Selective Endovascular Embolization

**DOI:** 10.1100/tsw.2007.147

**Published:** 2007-03-02

**Authors:** Sarel Halachmi, Amos Ofer, Eduard Gershin, Ahuva Engel, Shimon Meretyk

**Affiliations:** ^1^ The Department of Urology and Interventional Radiology, Rambam Medical Center, Haifa, Israel; ^2^ The Faculty of Medicine, Technion, Israel Institute of Technology, Haifa, Israel

**Keywords:** hemorrhage, complication, urological minimally invasive surgery, CT angiography, endovascular embolization

## Abstract

Minimally invasive urological procedures have gained in popularity and replaced open surgery in various urological procedures. Although considered minimally invasive, these procedures are not free from complications, and life-threatening hemorrhage may occur. Herein we describe 3 case series of patients who underwent minimally invasive urological surgeries that were complicated with bleeding. In all 3 patients we used super selective angiographic embolization to stop hemorrhage. Minimally invasive urological surgeries carry the risk of hemorrhage, and patients should be informed about this possibility. In hemodynnmic stable patients endovascular embolization allowed bleeding cessation with maximal preservation of the bleeding kidney tissue.

### INTRODUCTION

Renal or retroperitoneal hemorrhage may follow minimally invasive urological procedures, and can be life threatening. Prompt response with fluid and blood resuscitation is needed. Management is dependent on patient's homodynamic stability. For hemodynamically stable patients CT angiography (CTA) or angiography is indicated to identify and treat the bleeding source. The treatment of choice in relatively stable patient is super selective endovascular embolization to stop the bleeding immediately, while preserving as much as possible viable and functional tissue. Herein we describe three patients who underwent minimally invasive urological surgeries complicated with hemorrhage, and treated successfully by super selective endovascular embolization.

## CASE DESCRIPTION

### Case 1

A 65 years old morbidly obese man (weight 125Kg, height 175cm, BMI - 41) underwent left laparoscopic nephrectomy of an obstructed severely hydronephrotic non-functioning kidney. Vacuum drain left in the kidney bed drained daily between 150-400cc of cloudy fluid with high amylase content. The diagnosis of pancreatic tail injury was made, and the patient was treated conservatively with food restriction, intravenous fluids and alimentation, antibiotics and percutaneous drainage. The amount of the secretions slowly decreased to 50-100cc per 24hr.

On postoperative day 14, he abruptly bled 2 liters of fresh blood during a period of 30 minutes. The patient was resuscitated with rapid administration of fluids followed by 3 units packed red blood cells that achieved stabilization of his blood pressure and pulse. His homodynamic stability allowed us to postponed planes for explorative laparotomy, and the patient underwent angiographic computerized tomography (CTA), which demonstrated active bleeding from a small pseudoaneurysm of the left phrenic artery. Angiography with selective coil embolization stopped the bleeding. The patient remained hemodynamically stable throughout the procedure.

### Case 2

A 60 years old female diagnosed with left symptomatic uretero-pelvic junction obstruction, underwent left retrograde endopyelotomy due to recurrent severe left flank pain. Following the procedure the patient developed intermittent macroscopic hematuria with repeated drop in hemoglobin levels to values around 9 mg/dl. During a period of 2 weeks she received a total of 5 units of packed red blood cells. As she bled intermittently we initially tried to manage the case conservatively, however following the administration of the $5^{th}$ blood unit, she underwent renal angiography which showed a pseudoaneurysm of the left lower pole artery. This was treated successfully by selective coil embolization. Intra venous pyelography (IVP) performed two months later demonstrated good unobstructed function of the left kidney.

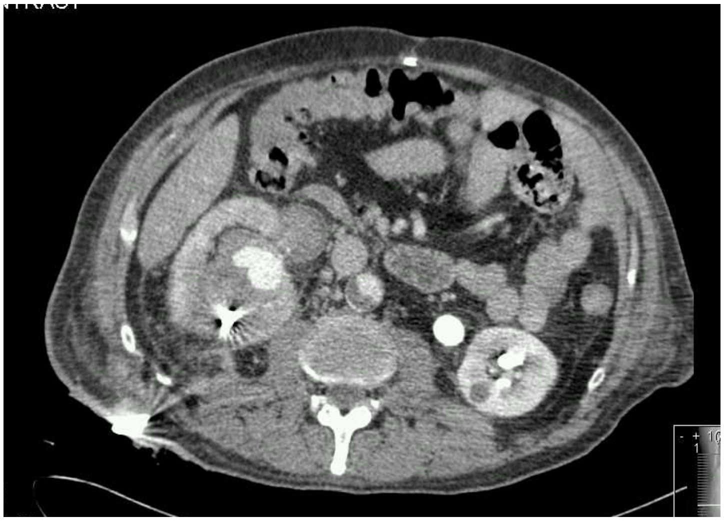

**FIGURE. 1:** CTA scan showing renal lower pole pseudoaneurysm (white arrow)

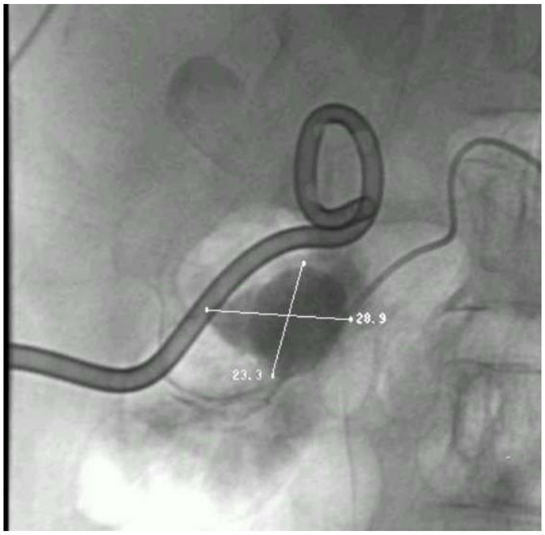

**FIGURE 2.** Selective renal angiography demonstrating 24 X 29 millimeter pseudoaneurysm of the lower pole segmental artery, just below the nephrostomy stent.

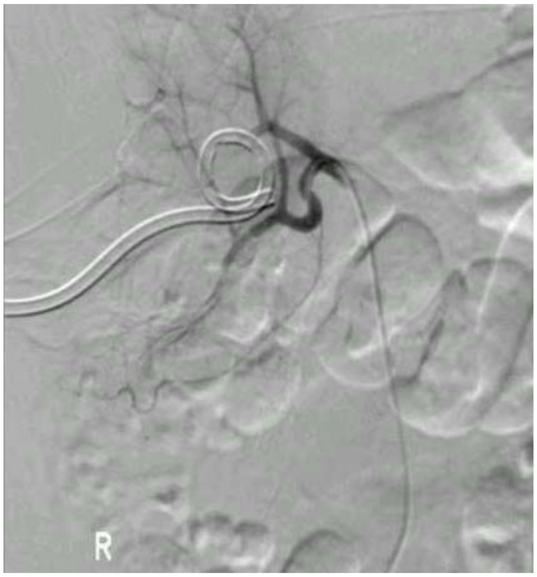

**FIGURE 3.** Post embolization film demonstrating the disappearance of the pseudo aneurysm. Vascular supply to the upper and lower pole was preserved.

### Case 3

A 72 years old man underwent right percutaneous nephrolithotripsy (PCNL) for staghorn calculi. Following the procedure he developed gross hematuria with blood clots causing intermittent obstruction and sever flank pain. The patient underwent CTA (Figure 1) that demonstrated active bleeding from a lower pole segmental artery pseudo aneurysm. The pseudo aneurysm was treated successfully by selective embolization with gelfoam (Figures 1,2,3). Nuclear scan performed 3 months later demonstrated good function of the upper pole, reduced function of the lower pole and non functioning renal tissue in the mid portion of the kidney.

### DISCUSSION

Minimally invasive procedures (endourological and laparoscopic) have changed the urological practice by reducing the need for large access surgical incisions, maximizing tissue preservation, shortening significantly the recovery and hospitalization period, lowering the need for narcotics, and reducing greatly the time to full recovery. Unfortunately, these procedures are not free from complications, and life threatening hemorrhage, although rare, is one of the most serious complications that may occur[1,2,3].

Management of bleeding patients includes volume resuscitation by rapid fluid and blood administration. Hemodynamically unstable patient should be rushed to the operating theater; however stable patients should undergo vascular radiographic evaluation. According to previous investigators and our own clinical experience the examination of choice in those situations is CT angiography[4,5,6]. Following the identification of the bleeding source, final management could be elected; the treatment varies between surgical resection of the bleeding organ, reconstruction of the damaged vessel/organ and angiographic embolization. Embolization has the advantage of being minimally invasiveness in nature with the ability of super selectivity blood vessels occlusion[7] allowing maximal preservation of viable non bleeding tissue.

Our 1st patient case demonstrated the advantage in the combination of the imaging modalities. The patient suffered from abdominal bleeding probably because of chemical vascular erosion due to pancreatic tail injury and pancreatic fluid spilage. It was very tempting to rush with the patient to the operating theater, although emergency operation in this morbidly obese patient with long standing pancreatic fistula, and severe metabolic depletion. Identification and treatment of the pseudoaneurysm of left phernic artery would have been very challenging to the surgeon and for the patient's ability to recover following 2nd major operation. Instead, CT angiography demonstrated the exact cause of bleeding allowing fast super selective endovascular embolization. Angiography alone might not have been more diagnostic than the CT as the source of bleeding was unknown.

Blood transfusion following PCNL may be needed in 5-12% of the patients[8]. Pseudo aneurysms or A-V fistulas occurs in 0.9 – 3%[9]. Tract hemorrhage may be treated by insertion of Kaye nephrostomy balloon catheter, however if the bleeding vessel is not compressed by the nephrostomy the next step should be radiological evaluation and angiography with possible embolization. Bleeding requiring transfusion may occur in 1-4% of patients undergoing Acucise endopyelotomy[10] due to polar renal artery hemorrhage. Most of the patients can be treated conservatively, however up to 1.5% continue to bleed demanding active management. In those who continue to bleed, angiography with embolization becomes the procedure of choice[7]. Traditionally angiography was the procedure of choice to diagnose and treat stable patients with suspected active bleeding. Recently the use of CTA for fast, accurate, and non invasive diagnosis of the cause and site of bleeding has been suggested[11,12,13]. CTA results allow the interventional radiologist to plan the endovascular treatment in advance and avoid unnecessary non-selective catheterizations. Super selective endovascular embolization allows rapid cessation of bleeding with maximal preservation of renal viable parenchyma. Despite of these advantages surgeons should aware for the possibility of angioembolization failure. In all of the described cases in parallel to the embolization attempt, operating theater scrub nurses and surgeons were ready for exploration in case of embolization failure.

In conclusion, renal or retroperitoneal hemorrhage following minimally invasive urological procedures can be life threatening. Prompt fluid and blood resuscitation is needed. If the patient is hemodynamically stable, radiological imaging using CTA is fast, non invasive and accurate for the diagnosis of the site and cause of bleeding, promoting pre planed super selective endovascular embolization, and preventing exploratory operation that may result in organ loss. Super selective endovascular embolization should be the procedure of choice being minimally invasive and allowing maximal preservation of renal parenchyma. Using these treatment options, fast and accurate diagnosis and treatment can be achieved, avoiding difficult emergency operations for severely ill patients.
